# Physical Activity and Outdoor Play of Children in Public Playgrounds—Do Gender and Social Environment Matter?

**DOI:** 10.3390/ijerph15071356

**Published:** 2018-06-28

**Authors:** Anne Kerstin Reimers, Stephanie Schoeppe, Yolanda Demetriou, Guido Knapp

**Affiliations:** 1 Institute of Human Movement Science and Health, Faculty of Behavioral and Social Sciences, Chemnitz University of Technology, 09111 Chemnitz, Germany; 2 School of Health, Medical and Applied Sciences, Physical Activity Research Group, Central Queensland University, Rockhampton 4702, Australia; s.schoeppe@cqu.edu.au; 3 Department of Sport and Health Sciences, Campus D–Uptown München, Technical University of Munich, 80992 München, Germany; yolanda.demetriou@tum.de; 4 Faculty of Statistics, Technische Universität Dortmund, 44221 Dortmund, Germany; guido.knapp@tu-dortmund.de

**Keywords:** physical activity, gender, outdoor play, playgrounds, children, direct observation, social environment

## Abstract

Background: Few studies have delved into the relationship of the social environment with children’s physical activity and outdoor play in public playgrounds by considering gender differences. The aim of the present study was to examine gender differences and the relationship of the social environment with children’s physical activity and outdoor play in public playgrounds. Methods: A quantitative, observational study was conducted at ten playgrounds in one district of a middle-sized town in Germany. The social environment, physical activity levels, and outdoor play were measured using a modified version of the System for Observing Play and Leisure Activity in Youth. Results: In total, 266 observations of children (117 girls/149 boys) between four and 12 years old were used in this analysis. Significant gender differences were found in relation to activity types, but not in moderate-to-vigorous physical activity (MVPA). The presence of active children was the main explanatory variable for MVPA. In the models stratified by gender, the presence of opposite-sex children was a significant negative predictor of MVPA in girls but not in boys. Conclusions: The presence of active children contributes to children’s physical activity levels in public playgrounds. Girls’ physical activity seems to be suppressed in the presence of boys.

## 1. Introduction

Playing and physical activity are essential for child development as they enable cognitive, physical, emotional, social, and motor learning, as well as well-being. The UN Convention on the Rights of the Child states that every child has a right to play. However, many children worldwide are not meeting physical activity guidelines [1] and have decreased opportunities for active outdoor play, which are both vital for a child’s healthy development [2]. In Germany, only 13% of girls and 17% of the boys aged between 4–17 years meet the WHO physical activity guidelines [3] recommending that children engage in at least one hour of moderate-to-vigorous physical activity (MVPA) a day [4]. Additionally, due to parental concerns about road safety and stranger danger, children nowadays have limited independent mobility (i.e., freedom to travel to places and play outdoors without adult supervision) and therefore less opportunity to play outside [5,6,7,8].

Physical and social environmental factors determine children’s physical activity and outdoor play [9]. With regard to the physical environment, play facilities such as playgrounds are important requirements for being physically active [10,11]. Therefore, to provide opportunities for children to be physically active, play, and interact with other children outdoors, accessible, usable, and safe physical activity and play spaces are needed [12,13]. Public playgrounds constitute important settings for children to play, experience and interact with their social and physical environment, recognize and test their own abilities, and develop social, physical, and motoric skills. Furthermore, playgrounds facilitate positive experiences such as fun, creation, self-efficacy, social interaction, creativeness, and physical ability [14] and may contribute to increased levels of energy expenditure in children [15].

Boys and girls often behave differently in regard to their physical activity and play behavior, as postulated in various theories on gender [16]. Empirical research studies have shown that boys and girls choose to play at and with different things in different ways and they differ in terms of their physical activity levels, types, and preferences [17]: For example, girls tend to be less physically active than boys overall [1,18] in school playgrounds [19,20,21], as well as out of school playgrounds [22]. In playgrounds, the following differences with regards to preferred play behaviors of girls and boys have been observed: For example, boys are more likely to play space-consuming and popular ball games such as football [14]. Krajicek playgrounds, which are newly created public playgrounds of the Richard Krajicek Foundation and which are “primarily aimed at offering play areas for team sports like soccer and basketball” [23], have been found to be more attractive for boys than girls. Additionally, Anthamatten et al. [24], as well as Floys et al. [25], found that boys utilize play fields, courts, or basketball areas more often than girls and exhibit higher levels of MVPA on hard-surfaced play areas and play equipment areas than girls in a schoolyard setting. Furthermore, boys and girls seem to be responsive to different playground features and qualities: in school playgrounds, girls experience a higher enjoyment level than boys for a number of activities like playing tag games, being active during play activities, walking, creating/making things, using imagination, climbing, sliding, hiding, sitting, and resting/relaxing [26].

The social environment (family, school, neighborhood) is an important predictor of children’s physical activity. As stated in Social Cognitive Theory [27], children’s behavior and behavioral choices are strongly affected by social models. Several modeling phenomena, each with different operating mechanisms, could be distinguished [28]. One phenomena of social modeling, which could be assumed as a predictor of children’s physical activity behavior, occurs when “others’ actions serve as social prompts, inducements or response-cuing effects” [28]. The same-sex imitation hypothesis assumes that children develop tendencies to prefer imitation of same-sex models [29]. More general, imitation of social models occurs as a function of credibility and relevance of the social models. For example, parents could be significant models for children: Parental modeling of active behaviors has been shown to positively influence children’s participation in physical activities such as outdoor play, sport, and walking [30,31]. Besides parental modeling, the presence of peer models being physically active is associated with an increase in physical activity [32,33]. In particular, in a playground setting, peer modeling and companionship/playing together may be especially relevant for the encouragement of physical activity and play [34]. Nevertheless, the presence of other persons can also lead to a decrease in physical activity or inhibit physical activity and how children play. For example, parental restrictions can lead to lower levels of children’s physical activity [35] and the presence of groups of teenagers can evoke feelings of fear, limiting physical activity behavior [36]. Furthermore, playground density is inversely related to physical activity in school playgrounds [20,37,38], which indicates that besides supportive effects of companionship and modeling, a lack of space per child prevents physical activity. While companionship is the precondition for many play activities like tag games, ball games, or seesaw, and while peer modeling and support could foster physical activity, a high number of children in the playground may hinder physical activity.

A large amount of studies exist that examined girls’ and boys’ physical activity and outdoor play in (pre)school playgrounds [39,40,41,42,43], but not in public playgrounds. Usage, physical activity, and play in public and in (pre)school playgrounds differ from one another. Children use school playgrounds obligatorily, while public playgrounds are visited during their leisure time voluntarily or with the encouragement of their parents/supervisors. In school playgrounds, children are surrounded with friends and familiar children attending the same school, whereas in public playgrounds, there is a greater variety of children and persons of different age groups (partially) unknown to the children. Additionally, the number of persons in public playgrounds varies more widely than in school playgrounds. Consequently, physical activity and outdoor play behavior in public playgrounds could differ from those found in schools.

The effects of the social environment on physical activity and play behavior in public playgrounds have rarely been examined by gender [10], especially in a non-school playground setting. Therefore, the aim of this study is to examine: (i) gender differences in physical activity, social play behavior, and preferred play equipment of children in public playgrounds; (ii) the relationship between social environmental factors (i.e., presence of active children, presence of adults) and the physical activity of children in public playgrounds; and (iii) whether this relationship is moderated by gender.

## 2. Method

### 2.1. Study Design

A quantitative, observational study was designed to examine weekday physical activity and outdoor play in children in playgrounds. The study was conducted in a district of Constance (i.e., Petershausen-West), a middle-sized town (83,000 inhabitants) in the south of Germany near the Swiss border. With approximately 3000 children and adolescents aged between 0–17 years [44] out of 22,000 total inhabitants of the district (Petershausen-West), it represents a district with a high proportion of young people. The district includes an area of 181.89 ha with a dense population (124.14 inhabitants per ha) in which a high proportion (83%) of the inhabitants are German and a low unemployment rate (3.6%). All ten playgrounds in the district of Petershausen-West were analyzed in this study. Detailed information on quality aspects of the playgrounds and playground users is provided elsewhere [45]. All procedures performed in this study were in accordance with the ethical standards of the Ethics Committee of the University of Konstanz (IRB15KN01) and with the 1964 Declaration of Helsinki and its later amendments. No informed consent was required from the playground users because only de-identified data was collected in this observational study.

### 2.2. Data Collection

A direct observation tool was used to assess the number of users in the playgrounds (all persons in the playgrounds including children, adolescents, and adults), children’s physical activity levels, activity types, group sizes, social interactions, and usage of playground equipment. The observation tool comprised items of the System for Observing Play and Leisure Activity in Youth (SOPLAY) [46], the System for Observing Children’s Activity and Relationships during Play (SOCARP) [47], and a newly developed item on children’s usage of playground equipment. This item captured whether and which playground equipment the observed child played with. Data collection took place from April to September 2015. The observers were trained by studying the SOCARP and SOPLAY protocols and by using the observer training videos [46,47]. The trained observers (*N* = 3) visited the playgrounds for one hour each between 10 a.m. and 8 p.m. to conduct direct observations based on a momentary time sampling procedure [48]. Thus, the observers visited the playground on a defined date and conducted the scans and individual observations. Scans were conducted to observe all playground users in the playgrounds and for individual observations, one child was randomly selected. During school days, the observations took place between 12 p.m. and 8 p.m., during the summer holiday (30th of July to 13th of September 2015) between 10 a.m. and 8 p.m.

The direct observation included scans of all playground users and observations of individual children. Up to five scans were conducted within one hour of observation (between the scans, individual observations were conducted as described below). During the scans, the observers let their eyes wander from one side to the other of the playground. To enable counting of the number of playground users and their physical activity levels, a mechanical counter was used [46]. Each scan encompassed six of these views over the playground. In the first view, the number of playground users within an age group was counted (the observers assigned the playground users to an age group by visual assessment). The following views were conducted to count the number of female children in relation to their physical activity level (sedentary, walking, or vigorous) and the female adolescents in relation to their physical activity level. Following this, the procedure was repeated for male users. In the present study, children were assigned to the children’s group when the estimated age was ≤12 years. Detailed information on physical activity levels of children in the playgrounds and playground usage is reported elsewhere [45].

For individual observations, up to four different children were observed within one hour of observation. For this, the observers randomly selected one child playing in the playground. Each child was observed for five minutes (=ten intervals of 30 s each time). Each interval included 10 s of observation following 20 s of recording. In order to keep the timeframes, a timer was used. During each hour of observation, the observation procedure was repeated up to four times. To control for time and observer effects, all playgrounds were equally examined by all observers and the hours were evenly distributed over the day (between 10 a.m. and 8 p.m.).

During the observations, the observers had a fixed position in the playground (e.g., a centrally located bench) from where they could observe the entire playground. To avoid reactivity by children, the observers behaved as unobtrusively as possible. In case of questions by parents or supervisors, the observers offered an informative letter containing details of the aims and procedure of the study, the responsible researchers and institutions, and data security measures. Observers also provided verbal information about their data collection in the playground and about the study, if requested by parents/supervisors or children.

### 2.3. Measures

#### 2.3.1. Children’s Physical Activity Behavior

A modified version of the System for Observing Children’s Activity and Relationships during Play (SOCARP) tool developed by Ridgers et al. [47] was used to assess a targeted child’s physical activity level, activity type, and group size in the playgrounds. It was modified to also assess usage of playground equipment. The physical activity level of the target child was recorded as sedentary (lying, sitting, standing), walking (walking, crawling), or vigorous (running, jumping, climbing) [47]. A child who was walking or engaging in vigorous physical activities was defined as being moderately-to-vigorously physically active (MVPA) [47]. Thus, every child was rated as being sedentary, moderately active, or vigorously active. Social group size was defined as the total number of persons in the group in which the target child was located (including the target child) and encompassed four categories: alone (without other persons), small (two to four persons), medium (five to nine persons), and large (10 and more persons). Activity type reflected the nature of the activity the target child engaged in the playground. To capture activity type, five categories were given based on SOCARP [47]: sports game (such as soccer, basketball), active games (such as hide-and-seek, chasing games), sedentary activities (such as reading, playing board or card games, sitting in the sandbox), locomotion (such as walking, running), and activities with playground equipment (such as swings, slides, etc.). The observer designated the type of activity with the best fit to the child. Furthermore, in the observation protocol, it was recorded whether the target child was using the following playground equipment: things to hang from, things to slide down, things to climb up, things to climb through things to stand/walk on, swings, carousels, see-saws, playing fields, or sandpits.

#### 2.3.2. Playground Density and Presence of (Active) Children and Adults in the Playgrounds

To capture the playground density and the number of (active) children in the playground within a defined time period by direct observation, a modified version of the SOPLAY observation tool [49] was applied. Using the direct observation tool, the number of users per age group (child, adolescent or adult) and the number of children featuring a physical activity level (sedentary, moderate, or vigorous) were examined separately for males and females. Gender and age group were rated from the observers through visual assessment. In line with the original SOPLAY instrument, sedentary behavior was defined and captured as lying, sitting, or standing. Walking was categorized as moderate physical activity and activities such as running, climbing, and jumping as vigorous activities. Children who were walking or engaging in vigorous physical activities were assigned to the moderate-to-vigorous physical activity group (number of active children). Variables of the number of present children, boys, girls, and adults were calculated. Furthermore, the number of active boys and girls, respectively, were calculated. Based on these variables, a variable of the number of same-sex children in the playground and a variable of the number of active same-sex children were calculated. Playground density was calculated by the quotient of the number of persons in the playground and playground size. Playground sizes were captured from the playground manual of the City of Constance [50].

#### 2.3.3. Confounding Factors

Daily weather variables (temperature during the hour of observation, rainfall) were recorded as potential confounding factors. Daily weather was captured using weather protocols provided by two German weather service webpages (www.wetterspiegel.de and www.wetterkontor.de).

#### 2.3.4. Statistical Analysis

Continuous variables are presented as the median with minimum and maximum values, as well as mean plus standard deviation (SD). For categorical variables, we report the absolute number and percentage. A comparison of categorical variables between girls and boys was assessed by a chi^2^ test or Fisher’s exact test. The main outcome variable is the percentage of being in MVPA during the up to 10 time intervals a single child was observed. We used a beta random variable to model the distribution of the percent values and used a multivariate regression analysis [51] to examine the effects of the independent variables (playground density, group size, active children, same-sex children, opposite-sex children, same-sex adults, opposite-sex adults and gender) on the outcome variable (MVPA). These independent variables cover relevant factors of the social environment. All beta regression models were conducted by adjusting for temperature and rainfall. Interaction effects between some independent variables and gender are analyzed and based on statistical significance included in the final overall model. Stratified models for boys and girls were also considered. The multivariate regression can only handle values in the open interval (0,1). Thus, we transformed the value 0 to 0.001 and the value 1 to 0.999. Consequently, on the logit scale, the response variable takes on values between−7 and 7. Two-sided *p*-values < 0.05 were considered as significantly different. No adjustment for multiple testing was done because the p-values should be interpreted in a descriptive manner. All analyses were conducted with SAS Software 9.4 (SAS Corporation, Cary, NC, USA).

## 3. Results

A total of 578 scans and 338 individual observations of children and adolescents in the playgrounds were conducted. Of these individual observations, 266 observations (117 girls/149 boys) for children between four and 12 years are used in this analysis. Each playground was observed 18–21 times. The size of the playgrounds ranged from 280 m^2^ to 3800 m^2^. On average, 6.4 (SD = 5.5) children were observed during a scan, and on average, 3.9 (SD = 3.3) children were moderately-to-vigorously physically active. Similar numbers of boys and girls were counted during the scans. Further descriptive information on social environmental measures is presented in Table 1. Further information on user characteristics of the playgrounds is presented in Table S1. 

Children’s physical activity and play behavior observed in the playgrounds are presented in Table 2. Within our study, most children were at least moderately active (63.2%), with more boys having higher proportions of MVPA than girls (66.4% vs. 59.0%). Boys and girls preferred similar playground equipment to play with: the only statistical significant difference was observed in regard to things to stand or walk on (*p* = 0.030). However, the numbers of children using a specific type of playground equipment were small, leading to three observations of children in the dimension “things to climb through” and up to 44 observations in the dimension “swings”. Approximately, one quarter of the observed children played alone (22.4% of girls and 24.8% of boys), and most observed children played in groups of two to four persons with no significant gender differences (72.4% of girls and 68.5% of boys). Only one girl and one boy were observed playing in a group of ten or more persons. Significant gender differences were found concerning the activity types: boys were more likely to engage in sports and active games (16.8%) than girls (4.3%), while girls were more likely to be walking/running (locomotion; 17.1% vs. 12.8%), to play on playground equipment (63.3% vs. 57.7%), or be sedentary (15.4% vs. 12.8%) than boys.

In the multivariate regression models (presented in Table 3), the interaction of gender with number of active children in the playground, as well as the number of opposite-sex children, predicted the MVPA of the observed children. The models stratified by gender revealed that the number of active children was positively associated with MVPA (Beta = 0.26; SE = 0.08; t = 3.09; *p* = 0.003) and the number of opposite-sex children was negatively associated with MVPA only in girls (Beta = −0.19; SE = 0.08; t = −2.45; *p* = 0.016). However, narrowly missing statistical significance, the number of active children was also positively associated with MVPA (Beta = 0.11; SE = 0.06; t = 1.74; *p* = 0.084) and the number of same-sex adults was negatively associated with MVPA (Beta = −0.26; SE = 0.14; t = −1.83; *p* = 0.069) in boys. Playground density and group size did not predict MVPA in any of the models. Besides the presented regression models which were adjusted for weather (rainfall and temperature), we also ran these models without adjustment. No substantial differences were found in these models, but model fit was better in the adjusted models. 

## 4. Discussion

The present study contributes to a better understanding of children’s physical activity and play in public playgrounds. It examines social environmental influences on these behaviors in detail and takes gender differences into account. Previous studies in this area primarily focused on physical activity behavior of children in school playgrounds [41,42] or often did not analyze gender influences in depth [52,53]. The results of this study showed that most children (63.2%) were moderately or vigorously active when playing in the playgrounds. Thus, the proportion of active children was considerably higher than in other studies conducted in public playgrounds or parks in the Netherlands, the US, and Australia [23,25,54]. Additionally, the present study revealed gender differences in the preferred type of activity and the usage of equipment.

Overall, no significant gender differences in physical activity levels were found, which is surprising because this finding is in contrast to many other studies [19,20,21,22,23,25]. However, preferred activity types differed between boys and girls, which is in line with the findings from other studies [19,20,21,53]: girls engaged more often in sedentary activities, locomotion, or activities on playground equipment, and boys were more likely to play sports or active games. Consequently, it seemed that the differences in types of activities did not lead to different levels of physical activity and that both boys and girls are actively playing in public playgrounds for most of the time.

Moreover, another study revealed that boys were more likely to play with materials associated with boys like cars and girls tended to occupy play spaces with activities related to girls like the doll corner in a kindergarten setting [15]. Furthermore, in a playground of a primary school from South Africa, girls were excluded from playing soccer with the boys [55]. The authors of these studies assume that there are stereotypical gender boundaries in play spaces that foster children to play according to the stereotypes. However, children crossing these gender boundaries have also been observed in these studies, but in the primary school study, the children not conforming to gender norms were often bullied or disapproved [55]. Thus, there might also be stereotypical gender boundaries preventing children from conducting activities not conforming to gender norms in public playgrounds.

Overall, none of the social environmental variables predicted MVPA in boys: Only in girls did the number of active children in the playground predict participation in MVPA and the number of boys being present was inversely related to MVPA. Playground density, group size, and presence of adults did not predict MVPA—neither in girls or boys. Thus, in the present study, the presence of active children who might serve as models or as active companions (companionship support), led to increased levels of MVPA of girls in public playgrounds. This finding is similar to the findings of an experimental study from Horne et al. [32] that was conducted in a primary school setting. In this study, physical activity levels increased during an intervention, in which fictional peer models acted as role models for physical activity [32]. However, in this study, the effects of the fictional role models were found in both genders. Additionally, in a study of children’s park-based physical activity in Durham NC, which did not account for interactions with gender, the presence of other active children had the strongest association with physical activity [25]. Consequently, the presence of other children being physically active could be a relevant factor fostering physical activity in children, especially in girls.

Furthermore, in the present study, the presence of same-sex children did not predict MVPA in boys or girls, respectively, indicating that the gender of models or play companions was irrelevant. This contradicts the same-sex hypothesis published by Bussey and Perry [29], which postulates that children tend to imitate models of their own sex. However, girls were less likely to be active when more boys were present at the playgrounds. The presence of boys could probably hinder physical activity in girls playing in playgrounds, given that boys tend to use larger spaces when playing [56]. Furthermore, boys may exclude girls while playing in the playgrounds [55,57,58] and therefore, reduce girls’ opportunities to take part in active games like ball games or playing tag. Supporting this presumption, in the present study, it has also been shown that girls were less often playing active or sports games than boys. For example, as shown by McGuffey and Rich [59], boys could have used hegemonic masculinity to regulate girls’ boundaries in outdoor play and to keep them out of the boys’ domains like sport games. However, dismissive behavior and social interactions have not been analyzed in the present study. Hence, we do not know the mechanisms that prevented girls from being physically active when more boys were present.

Previous research showed that playground density was inversely associated with physical activity levels of children in school playgrounds [20,37,38,39]. However, these studies took place during recess and playground density seems to be much higher during recess compared to public playgrounds: The mean density in the present study was 0.008 children per m^2^, while in the study of Cardon et al. [20], it was 0.15. In the study of D’Haese et al. [37] the play space per child was 12.18 m^2^, by Ridgers et al. [38] it was 6.2 m^2^, and by Van Cauwenberghe et al. [39] it was 7.4 m^2^, resulting in playground densities of 0.082, 0.161, and 0.135 children per m^2^, respectively. Consequently, the lower playground density in public playgrounds could offer better opportunities for being physically active than in school playgrounds during recess. Boys and girls were organized in similar group sizes. Neither boys nor girls had more children around them to play with.

Nonetheless, we did not analyze the types of interaction in the playgrounds. Consequently, we do not know if the present children or adults were interacting with the observed child in a supportive or restrictive way. For example, besides motivating or inviting the child to play with each other, the adults could have restricted the child’s activities by prohibition on the grounds of fear, as shown in other studies summarized in a meta-study by Lee et al. [60]. Moreover, other children could have bullied the observed child physically or verbally so that it retired and was less active: For example, an ethnographic study of children aged 6–10 in South Africa showed that boys were bullying girls and boys who did not confirm to gender norms and excluded them from their soccer play [55]. Another observational study showed that bullying was a very common behavior in playgrounds [61].

### Strengths and Limitations

The strength of the current study is the analysis of physical activity and social play behavior of children in neighborhood playgrounds and time away from school. This study also takes into account gender differences and provides real-time data by observation of physical activity and social environmental aspects in public playgrounds. A comprehensive data set of observations of play and physical activity behavior of individual children and their social environmental surroundings has been collected. 

This study also has some limitations. Firstly, only 10 playgrounds in one district of a middle-sized German town were assessed, only on weekdays and in the summertime of a single year. Furthermore, the number of boys and girls using playground equipment was somewhat low (e.g., things to climb through *N* = 3). Thus, these results are limited in their generality. Secondly, misjudgments could have occurred due to difficulties in estimating the age and gender of the playground users by subjective assessment using human observers. And as we did not capture the personal data of the observed children, we were not able to analyze the relevance of socio-economic status or migration backgrounds. Thirdly, the observations might have affected the children and playground users and their behavior. Finally, as we conducted an observational study with a quantitative observation protocol capturing presence and activity levels of playground users, we did not gather information on the interaction behavior of the social environment. Hence, we do not know if the social environment supported or hindered the physical activity and play behavior of the observed children.

## 5. Conclusions

The current study showed that the presence of active children contributed to girls’ physical activity levels in playgrounds. Additionally, girls’ physical activity was suppressed when boys were present in the playground, indicating that girls could have been distracted or bullied by boys simultaneously playing in the playgrounds [55]. Potentially, public playgrounds should provide more spaces for being physically active—especially for girls.

The results of the present study indicated gender stereotypical play behavior in public playgrounds. It seemed to be necessary to break down these stereotypes to achieve more multifaceted play opportunities for boys and girls, respectively. However, physical activity levels in public playgrounds seem to converge between boys and girls and differences decrease. Hence, public playgrounds have the potential to foster physical activities in boys and girls and thereby contribute to child health. During weekdays, frequently visited public playgrounds like the playgrounds in the study district can offer opportunities to play with other children and to engage in moderate or vigorous physical activities together with other children.

In summary, playgrounds seem to be places for boys and girls to be physically active and to interact with other children whereof they could benefit with respect to their physical activities and resulting health outcomes. 

## Figures and Tables

**Table 1:** Information on Social Environmental Variables.

Variable	Definition	*N*	Median (Min, Max)	Mean (SD)
Playground density	number of persons on the playground divided by the area of the playground	231	5 × 10^−3^ (7.7 × 10^−4^, 7.3 × 10^−2^)	8.1 × 10^−3^ (9.2 × 10^−3^)
Number of children in the playgrounds	total number of children observed in the playground	231	5 (1, 36)	6.4 (5.5)
Number of boys in the playgrounds	number of boys observed in the playground	231	2 (0, 18)	3.2 (3.0)
Number of girls in the playgrounds	number of girls observed in the playground	231	3 (0, 19)	3.2 (3.1)
Number of active children	number of children with MVPA observed in the playground	189	3 (0, 25)	3.9 (3.3)
Number of active boys	number of boys with MVPA observed in the playground	214	1 (0, 11)	1.9 (1.8)
Number of active girls	number of girls with MVPA observed in the playground	206	1 (0, 14)	1.8 (1.9)
Number of male adults	number of male adults observed in the playground	231	2 (0, 15)	2.3 (2.3)
Number of female adults	number of male adults observed in the playground	231	0 (0, 5)	0.7 (0.9)

Note: *N* = number of observations; this table contains only observations when the observed child was already present at the beginning of the scan. This procedure led to the loss of 35 observations.

**Table 2:** Physical Activity and Play Behavior of Children Observed in the Playgrounds.

Variable	Overall	Girls	Boys	*p*-Value
	*N* (%)	*N* (%)	*N* (%)	(Chi-Square Test)
Physical activity level				
Sedentary	98 (36.8)	48 (41.0)	50 (33.6)	0.252
Moderate	60 (22.6)	28 (23.9)	32 (21.5)	
Vigorous	108 (40.6)	41 (35.0)	67 (45.0)	
MVPA (moderate and vigorous)	168 (63.2)	69 (59.0)	99 (66.4)	
Activity type				
Sports games	8 (3.0)	0 (0)	8 (5.4)	0.019 *
Active games	22 (8.3)	5 (4.3)	17 (11.4)	
Sedentary activities	37 (13.9)	18 (15.4)	19 (12.8)	
Locomotion	39 (14.7)	20 (17.1)	19 (12.8)	
Activities on playground equipment	160 (60.2)	74 (63.3)	86 (57.7)	
Group size				
Alone (without other persons)	63 (23.8)	26 (22.4)	37 (24.8)	0.712
Small (2 to 4 persons)	186 (70.2)	84 (72.4)	102 (68.5)	
Medium (5 to 9 persons)	14 (5.3)	5 (4.3)	9 (6.0)	
Large (10 and more persons)	2 (0.8)	1 (0.9)	1 (0.7)	
Usage of playground equipment				Fisher’s exact test
Things to hang from	14 (5.3)	6 (5.1)	8 (5.4)	1
Things to slide down	33 (12.5)	10 (8.6)	23 (15.5)	0.095
Things to climb up	30 (11.3)	15 (12.8)	15 (10.1)	0.560
Things to climb through	3 (1.1)	0 (0)	3 (2.0)	0.258
Things to stand/walk on	15 (5.7)	11 (9.4)	4 (2.7)	0.030 *
Swings	44 (16.6)	24 (20.5)	20 (13.5)	0.138
Carousels	8 (3.0)	5 (4.3)	3 (2.0)	0.308
See-saws	4 (1.5)	1 (0.9)	3 (2.0)	0.633
Playing fields	8 (3.0)	2 (1.7)	6 (4.0)	0.472
Sandpits	18 (6.8)	11 (9.4)	7 (4.7)	0.147

Note: * *p* < 0.05.

**Table 3:** Effects of the Social Environment on Moderate-to-Vigorous-Physical Activity in Children’s Playgrounds.

Explanatory Factors	Overall				Girls				Boys			
	Beta	SE	t	*p*	Beta	SE	t	*p*	Beta	SE	t	*p*
Intercept	1.35	0.38	3.55	0.001 *	0.69	0.60	1.14	0.258	1.97	0.48	4.10	<0.001 *
Playground density	−4.37	11.19	−0.39	0.697	−17.24	18.29	−0.94	0.348	7.31	14.39	0.51	0.613
Group size												
Medium (5 to 9 persons)	−0.33	0.44	−0.74	0.460	−0.41	0.75	−0.55	0.580	−0.22	0.56	−0.40	0.688
Small (2 to 4 persons)	−0.26	0.21	−1.25	0.212	−0.29	0.32	−0.89	0.376	−0.23	0.26	−0.87	0.384
Alone (without others) (ref.)												
Active children	0.10	0.06	1.76	0.080	0.26	0.08	3.09	0.003 *	0.11	0.06	1.74	0.084
Same-sex children	−0.05	0.05	−1.10	0.273	−0.04	0.08	−0.47	0.642	−0.07	0.06	−1.13	0.260
Opposite-sex children	0.01	0.06	0.22	0.823	−0.19	0.08	−2.45	0.016 *	0.02	0.06	0.40	0.693
Same-sex adults	−0.07	0.07	−0.96	0.338	−0.05	0.09	−0.59	0.558	−0.26	0.14	−1.83	0.069
Opposite-sex adults	−0.04	0.06	−0.64	0.526	0.10	0.15	0.69	0.491	−0.03	0.07	−0.43	0.665
Gender	−0.27	0.26	−1.05	0.297								
Gender * Active children	0.17	0.08	2.21	0.028 *								
Gender * Opposite-sex children	−0.21	0.08	−2.50	0.013 *								
Scale	0.97	0.08			0.83	0.09			1.23	0.14		

Note: SE = standard error; ref. = reference; * *p* < 0.05; models were adjusted for rainfall and temperature; Data was weighted using the complex sample procedure.

## Supplementary Materials

The following are available online at http://www.mdpi.com/1660-4601/15/7/1356/s1. Table S1: Information on user characteristics of the playgrounds.
