# Supplementary material for: Physical Activity and Outdoor Play of Children in Public Playgrounds—Do Gender and Social Environment Matter?
Source: Int J Environ Res Public Health. 2018 Jun 28;15(7):1356. doi: 10.3390/ijerph15071356 (PMC6069007; doi:10.3390/ijerph15071356)
Supplement: Supplementary file 1 [file ijerph-15-01356-s001.pdf]

Table S1. Information on user characteristics of the playgrounds.

**Variable**		**Playground no.**									
		1	2	3	4	5	6	7	8	9	10
Playground density	*N*	34	38	5	15	46	1	4	35	33	20
	M	22,5 x 10^{-3}	4,7 x 10^{-3}	14,6 x 10^{-3}	4,7 x 10^{-3}	4,4 x 10^{-3}	1,7 x 10^{-3}	1,2 x 10^{-3}	9,3 x 10^{-3}	6.2 x 10^{-3}	2,9 x 10^{-3}
	SD	15,7 x 10^{-3}	2,5 x 10^{-3}	3,9 x 10^{-3}	1,8 x 10^{-3}	3,0 x 10^{-3}	.	0,5 x 10^{-3}	4,0 x 10^{-3}	5,4 x 10^{-3}	1,3 x 10^{-3}
Number of children in the playground	*N*	34	38	5	15	46	1	4	35	33	20
	M	6.94	6.29	2.60	3.67	10.87	5.00	1.75	6.71	4.18	2.80
	SD	5.68	3.36	0.89	1.54	8.35	.	0.96	3.27	2.92	1.51
Number of boys in the playground	*N*	34	38	5	15	46	1	4	35	33	20
	M	3.15	3.00	1.80	2.07	5.39	1.00	0.75	3.54	2.30	1.20
	SD	3.15	2.29	0.45	1.62	4.03	.	0.50	1.79	2.51	1.11
Number of girls in the playground	*N*	34	38	5	15	46	1	4	35	33	20
	M	3.79	3.29	0.80	1.60	5.48	4.00	1.00	3.17	1.88	1.60
	SD	3.13	1.77	0.45	1.30	5.09	.	1.15	1.98	1.14	0.94
Number of active children	*N*	32	35	4	10	40	1	3	28	23	13
	M	4.72	3.69	1.75	1.80	5.98	3.00	1.00	3.68	2.74	1.08
	SD	4.40	2.63	0.50	0.79	3.53	.	1.00	2.31	2.22	1.32
Number of active boys	*N*	33	37	5	13	45	1	3	34	27	16
	M	2.09	1.54	1.20	1.08	3.07	1.00	0.33	2.06	1.74	0.63
	SD	2.11	1.39	0.84	0.76	2.24	.	0.58	1.54	1.58	0.89
Number of active girls	*N*	33	36	4	12	41	1	4	29	29	17
	M	2.61	2.06	0.25	0.58	2.68	2.00	0.75	1.59	0.90	0.59
	SD	2.78	1.60	0.50	0.79	2.16	.	0.96	1.30	0.90	0.94
Number of adults	*N*	34	38	5	15	46	1	4	35	33	20
	M	8.94	4.03	1.20	2.47	5.72	0.00	1.25	2.66	1.58	1.05
	SD	6.28	2.14	0.45	1.19	3.76	.	0.50	1.63	2.18	1.36

Note: *M* = Mean; *N* = Number of observations; *SD* = Standard deviation
